# Identification of Cellular Pathogenicity Markers for SIL1 Mutations Linked to Marinesco-Sjögren Syndrome

**DOI:** 10.3389/fneur.2019.00562

**Published:** 2019-06-14

**Authors:** Christian Gatz, Denisa Hathazi, Ute Münchberg, Stephan Buchkremer, Thomas Labisch, Ben Munro, Rita Horvath, Ana Töpf, Joachim Weis, Andreas Roos

**Affiliations:** ^1^Institute of Neuropathology, RWTH Aachen University Hospital, Aachen, Germany; ^2^Leibniz-Institut für Analytische Wissenschaften—ISAS—e.V., Dortmund, Germany; ^3^Department of Clinical Neuroscience, University of Cambridge, Cambridge, United Kingdom; ^4^International Centre for Life, Institute of Genetic Medicine, Newcastle upon Tyne, United Kingdom; ^5^Pediatric Neurology, Faculty of Medicine, University Childrens Hospital, University of Duisburg-Essen, Essen, Germany

**Keywords:** SIL1-interactome, Marinesco-Sjögren syndrome biomarkers, SIL1 missense mutation, POC1A, FAM134B, centrosome

## Abstract

**Background and objective:** Recessive mutations in the *SIL1* gene cause Marinesco-Sjögren syndrome (MSS), a rare neuropediatric disorder. MSS-patients typically present with congenital cataracts, intellectual disability, cerebellar ataxia and progressive vacuolar myopathy. However, atypical clinical presentations associated with *SIL1* mutations have been described over the last years; compound heterozygosity of SIL1 missense mutations even resulted in a phenotype not fulfilling the clinical diagnostic criteria of MSS. Thus, a read-out system to evaluate reliably the pathogenicity of amino acid changes in *SIL1* is needed. Here, we aim to provide suitable cellular biomarkers enabling the robust evaluation of pathogenicity of SIL1 mutations.

**Methods:** Five *SIL1* variants including one polymorphism (p.K132Q), three known pathogenic mutations (p.V231_I232del, p.G312R, and p.L457P) and one ambiguous missense variant (p.R92W) were studied along with the wild-type proteins in Hek293 *in vitro* models by cell biological assays, immunoprecipitation, immunoblotting, and immunofluorescence as well as electron microscopy. Moreover, the SIL1-interactomes were interrogated by tandem-affinity-purification and subsequent mass spectrometry.

**Results:** Our combined studies confirmed the pathogenicity of p.V231_I232del, p.G312R, and p.L457P by showing instability of the proteins as well as tendency to form aggregates. This observation is in line with altered structure of the ER-Golgi system and vacuole formation upon expression of these pathogenic SIL1-mutants as well as the presence of oxidative or ER-stress. Reduced cellular fitness along with abnormal mitochondrial architecture could also be observed. Notably, both the polymorphic p.K132Q and the ambiguous p.R92W variants did not elicit such alterations. Study of the SIL1-interactome identified POC1A as a novel binding partner of wild-type SIL1; the interaction is disrupted upon the presence of pathogenic mutants but not influenced by the presence of benign variants. Disrupted SIL1-POC1A interaction is associated with centrosome disintegration.

**Conclusions:** We developed a combination of cellular outcome measures to evaluate the pathogenicity of *SIL1* variants in suitable *in vitro* models and demonstrated that the p. R92W missense variant is a polymorphism rather than a pathogenic mutation leading to MSS.

## Introduction

Marinesco-Sjögren syndrome (MSS) was established as an entity by the Romanian neurologist Georges Marinesco in 1931 based on the description of four individuals from a single family presenting with ataxia, cataracts, intellectual disability, and myopathy. This was followed by Torsten Sjögren who further defined the original four cases as well as 10 additional patients. Afterwards, cerebellar atrophy was described as part of the phenotypic spectrum (1). In 2005, two groups independently reported recessive mutations in the *SIL1* gene as the major genetic cause for MSS (2, 3). *SIL1* encodes a co-chaperone for the major Endoplasmic Reticulum (ER)-resident chaperone BiP and thus controls a variety of BiP-dependent functions such as protein folding (4). Hereby, SIL1 acts as a N-linked glycoprotein equipped with an N-terminal ER targeting sequence and a C-terminal ER retention signal. SIL1 dimerises at the N-terminus into a clamp-like configuration that interacts with the BiP ATPase domain and causes substrate release of BiP via the release of ADP (5).

The “clinical triad” of MSS is defined by presence of bilateral cataracts, ataxia, and myopathy whereas intellectual disability can manifest with very varying degree or can even be absent (6). So far, a clear genotype-phenotype correlation does not exist, and the phenotypical presentation becomes even more complex by the description of additional features: pectus carinatum and bilateral clinodactyly in a patient with a homozygous large indel in the 5′UTR (7), Dandy-Walker malformations in a Chinese family with a non-stop mutation (8) and associated motor neuronopathy with a bradykinetic movement disorder in a 5-year-old child, suggesting an intriguing continuum between neurodevelopmental and neurodegenerative multisystem disorders intricately linked in the same cellular pathways (9). Moreover, the phenotypical variation has become even more complex by the first report of compound heterozygous *SIL1* missense mutations causing a neurological phenotype without signs of myopathy or cataracts but including spastic paraplegia, thus not fulfilling the clinical diagnostic criteria for MSS (10). Interestingly, the number of detected *SIL1* variants (*n* = 488; ExAC http://exac.broadinstitute.org/) as of 11/2018 includes 147 missense and 7 non-sense variants with various effects on the phenotype. Notably, these missense mutations also include the p.R92W variant of SIL1, described as segregating with the phenotype in a consanguineous family from Pakistan (11) but reported in ExAC with an allele frequency of nearly 4% in south Asian countries suggesting a polymorphic character. Given that *(i)* the MSS phenotype can present with prominent additional clinical features or characteristic features of the “clinical triad” can be absent, *(ii)* missense mutations may have a detrimental effect (10) and *(iii)* the detection of new *SIL1* sequence variants ever increasing in number, the need to classify the pathogenicity is indicated. Here we introduce an *in vitro* system designed to examine the consequences of *SIL1* mutations at the protein level, focussing on the stability of the variant SIL1 proteins, the morphology of cellular organelles, the build-up of aggregated proteins, the activation of proteolysis and on the SIL1-interactome. This procedure has allowed to differentiate between benign and pathogenic variants (mostly due to amino acid changes).

## Materials and Methods

### Generation of SIL1-TAP and SIL1-HA Expression Constructs

Using the SIL1-TAP expression construct (pcDNA5/FRT/TO vector) (12) missense variants (listed below) as well as one deletion variant of recombinant human *SIL1* were generated by site-directed mutagenesis. To this end, the QuikChange Site-Directed Mutagenesis kit (Stratagene) was used according to the manufacturer's instructions. Primer sequences are available on request. All constructs were verified by sequencing the entire coding region of the inserts.

The ORF of the human *SIL1* cDNA was cloned in-frame into the carboxy-terminal hemagglutinin (HA) tag containing pRC/CMV expression vector. The same missense changes as for the pcDNA5/FRT/TO vector systems were introduced by site-directed mutagenesis using the QuikChange Site-Directed Mutagenesis kit (Stratagene). Primer sequences are available on request. All constructs were verified by sequencing the entire coding region of the inserts.

The following *SIL1* variants were selected and generated as expression systems based on the following reasons:

#### p.R92W

A missense variant of ambiguous pathogenic character: reported to cause MSS in a consanguineous Pakistani family (11) but present in ExAC with an allele frequency of nearly 4% and hereby also found to be homozygous in south Asian populations. Evaluation of its pathogenicity was one aim of the present study to address the suitability of our introduced *in vitro* system along with different tests.

#### p.K132Q

A missense variant of benign character (polymorphism) serving as a control. which should give the same results as the wild-type form of the SIL1 protein.

#### p.V231_I232del

A deletion variant with well-known pathogenicity based on stability of the protein as described previously by immunoblot and proteomics-based studies of MSS-patient-derived immortalized lymphoblastoid cells (6, 13).

#### p.G312R

A missense variant of well-known pathogenicity based on stability of the protein as described previously by immunoblot- and proteomics-based studies of MSS-patient derived immortalized lymphoblastoid cells (6, 13).

#### p.L457P

A missense variant with well-known pathogenicity *in vitro* tested in COS7 cells and associated with clinical presentation of MSS (14).

### Cell Culture and Treatments:

Using the six pcDNA/FRT/TO-based expression constructs for SIL1-TAP (wild-type and five variants), stable inducible Hek293-TRex cell lines were generated as described previously (12). Cells were cultured in Dulbecco's Modified Eagle's medium (DMEM; Sigma-D5546) supplemented with 10 fetal calf serum (FCS; Sigma- F2442). Transient transfection of pRC/CMV-based constructs for SIL1-HA wildtype and variants was performed utilizing Lipofectamine 2000 (Thermo Fisher Scientific) according to the manufacturer's instructions.

### Immunoprecipitation of the SIL1 Variants:

As SIL1 can act as a dimer (5), the ability to form protein dimers was addressed for the five protein variants of SIL1. Dimerization of the wildtype-protein was examined as a positive control to demonstrate functionality of the assay. The generated stable inducible Hek293-TRex cell lines (grown on 10 cm dishes) were additionally transfected with pRC/CMV-based constructs for SIL1-HA wildtype and variants and were collected 8 h post-transfection. Afterwards, cells were lysed in 500 μl of a buffer containing 1 mM EDTA (Sigma-Aldrich), 10% (v/v) glycerol (Roth), 20 mm HEPES (pH 7.5; Sigma-Aldrich), 150 mM NaCl (Sigma-Aldrich) and 0.8% (v/v) NP-40 (Roth) as well as 1 × Complete Protease Inhibitor Cocktail (Roche) for 1 h on ice. Next, centrifugation (1.500 rpm) of the lysates was performed at 4°C for 10 min to separate the protein extracts from cell debris. Protein concentrations were determined using the Pierce BCA protein assay kit (Thermo Fisher) according to manufacturer's instructions. Immunoprecipitation of the different HA-tagged forms of the SIL1 proteins via the TAP-tagged forms was carried out as described previously (12) using 100 μg of total protein extract.

For each experiment, cells overexpressing the solely the C-terminal TAP-Tag were included to exclude unspecific binding of the HA-Tagged versions of the SIL1 protein to the TAP-tag. All co-immunoprecipitation experiments were carried out three times.

### Tandem-Affinity Purification of SIL1-Interactome and LC-MS/MS-Based Identification of Binding Partners

Interaction screening [tandem-affinity-purification (15) and subsequent mass spectrometry] of SIL1 wildtype protein as well as the five variant forms was carried out as described previously (6, 12) For each experiment, three independent biological replicates were analyzed.

### Investigation of Cellular Fitness

Cellular fitness of Hek293-TRex cell lines overexpressing the wildtype SIL1 protein as well as of cells overexpressing the five variants was addressed by utilizing the WST-1 assay (Roche), according to the manufacturer's instructions. Cells treated with H_2_O_2_ were included as a positive control of decreased cellular fitness to demonstrate functionality of the assay. Moreover, cells overexpressing the TAP-Tag only were included to demonstrate that the presence/expression of the TAP-tag has no effect on cellular fitness. The relative absorption in cells overexpressing the SIL1 wildtype protein was considered as 100% of cellular fitness.

### Native-PAGE, SDS-PAGE and Western Blotting:

Native-PAGE was carried out as described in a protocol available online (http://www.assay-protocol.com/molecular-biology/electrophoresis/native-page) and the immunoblot studies were performed as described previously (16). The following antibodies were used:

### Immunofluorescence:

Distribution of SIL1 wildtype protein as well as of the five different variants was addressed in the stable inducible Hek293-TRex cell lines: the respective cell lines (2 × 10^5^ cells) were plated onto 24-well plates on coverslips 12 h prior to induction of expression with doxycycline as described before (12). Beforehand, coverslips were coated with poly-L-lysine (Sigma-Aldrich) for 1 h at room and afterwards washed twice with ultra-pure water. Cells were fixed with 4% paraformaldehyde in PBS pH 7.4 for 20 min at room temperature. Afterwards, cells were washed 3 times with ice-cold PBS. For permeabilization, coverslips were incubated for 10 min in PBS containing 0.25% Triton X-100 (Roth). In the following step, cells were washed in PBS 3 times for 5 min. For blocking and immunostaining, coverslips were incubated in 1% BSA (in PBS+ 0.1% Tween 20) for 45 min and afterwards exposed to the anti-TAP antibody (Sigma-Aldrich) 1:100 diluted in 1% BSA in PBST in a humidified chamber overnight at 4°C. Afterwards, coverslips were washed 3 times in PBS (5 min each washing step) and then incubated with the secondary antibody (goat anti-rabbit Alexa Fluor® 568, Abcam) diluted 1:5,000 in 1% BSA-PBS for 1 h at room temperature in the dark. Next, three washing steps times in PBS (5 min each washing step) were carried out. Finally, cells were mounted with a drop of mounting medium (Thermo Fisher) and coverslips were sealed with nail polish to prevent drying and movement under microscope. For analysis of immunoreactivity, the Axiovert 200 M microscope from Zeiss (240525) was used. For each SIL1 variant as well as for the wildtype proteins 50 different cells were analyzed with more than 70% of cells giving similar results, respectively.

For co-localization studies of SIL1, Hek293 cells were permeabilized by incubation with 200 μL PBS buffer containing 0.1% Triton X-100 for 10 min and subsequently washed with PBS buffer. For blocking cells were incubated with 200 μL of PBS containing 0.1% TWEEN20 and 1% BSA for 30 min. Afterwards, cells were covered with 200 μL of blocking solution containing the respective combinations of both primary antibodies [α-CASQ1 (Sigma: C0618-200UL), α-FAM134B (Genetex: GTX46621), α-POC1A (Thermo Fisher Scientific: PA5-49028), α-SIL1 (Abcam: ab5639) dilution 1:300 each], incubated for 90 min and then washed with PBS. Secondary antibodies were diluted in 1% BSA (dilution 1:500 for Alexa488 probe, 1:300 for Alexa555 probe). Cells were covered with 200 μl of this antibody-solutions and incubated for 1 h in the dark. Next, antibody solution was removed, and samples were washed with BSA before the coverslips were directly mounted with fluorescent mounting medium (“Prolong Gold antifade reagent with DAPI” Invitrogen) and allowed to dry overnight (at 6°C). Fluorescence measurements were carried out with a modified Leica TCS SP8 CARS laser scanning Microscope using either a 20x objective (HC PL APO CS2 20x/0.75 DRY) or a 63x objective (HC PL APO CS2 63x/1.20 WATER). Images were acquired with a resolution of 2048 × 2048 pixel with a step size of 286 nm for 20x magnification and of 91 nm for 63x magnification. Fluorescence measurements on each position were performed sequentially using laser excitation at 488 nm and detection at 500–540 nm with a hybrid detector (LeicaHyD), excitation at 561 nm and detection at 566–620 nm with a HyD and excitation at 405 nm with detection at 425–500 nm using a PMT. All data processing was carried out using Matlab R2015a. Due to variations in fluorescence intensity between the samples as well as between the different dyes data were preprocessed for better comparability. To reduce background, noise data points having < 2% of intensity were set to this lower threshold value. To account for cosmic spikes, the upper threshold was set so that a maximum of 0.1% of all data points above background showed fluorescence intensity above this limit. Intensities above the upper threshold were set to this value. Images were then rescaled to full range (8bit). For calculation of co-localization values in each channel, all data points exceeding an intensity value of 20% were determined. Data points that are above this value in both channels were considered to be co-localizing. The ratio of co-localization is calculated from the number of co-localizing points divided.

### Electron Microscopy

Preparation of Hek293-TRex cell lines and subsequent transmission electron microscopic studies were carried out as described previously (12, 16). For each SIL1 variant as well as for the wildtype protein, at least 50 cells were analyzed showing similar (patho)morphology of subcellular organelles/structures.

## Results

### Literature and *in silico*-Based Evaluation of the Pathogenicity of Selected SIL1-Variants

**Figure 1:**
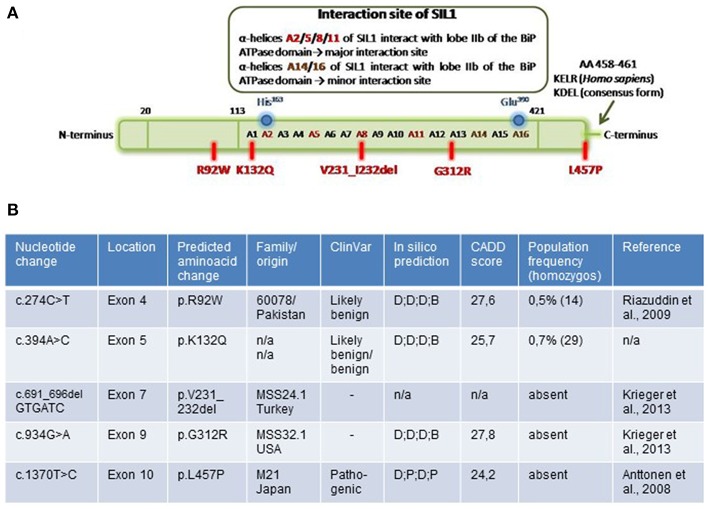
Overview of investigated amino acid changes of SIL1. **(A)** Localization of SIL1 amino acid changes in the entire SIL1 protein. Schematic figure is modified from Yan et al. (5). **(B)** Table summarizing the *SIL1* variants by providing information regarding the corresponding nucleotide change/ deletion in different exons within the *SIL1* gene as well as origin of the respective MSS-patient and results of *in silico*-based testing of pathogenicity by “CADD score” and “ClinVar” as well as by “SIFT“, “PluPhen2“, “MutationTaster“ and “FATHMM” (summarized as in silico prediction). D, damagingl; P, pathogenic; B, benign; T, tolerated.

We chose one known polymorphism (p.K132Q), three notoriously pathogenic mutations (p.V231_I232del, p.G312R, and p.L457P) and p.R92W, a variant that was previously described as a pathogenic missense mutation (11). Notably, the two missense variants pR92W and p.K132Q are present in the control population with minor allele frequencies (MAF) of 0.5 and 0.7%, respectively, and in 29 and 72 healthy individuals in homozygous state. On the other hand, p.G312R is absent from a control population of >125,000 individuals (ExaC, http://exac.broadinstitute.org) and p.L457P is found in one allele count (MAF: 0.0004%) (http://gnomad.broadinstitute.org). The sites of the different variants/ mutations are depicted in Figure 1A. By affecting α-helix A8 of the SIL1-protein, the p.V231_I232del mutation directly impacts on the physiological SIL1-BiP interaction lobe IIb of the BiP-ATPase domain (major interaction site). Although the polymorphic variant p.K132Q and the reported pathogenic missense mutant p.G312R are also localized within the SIL1-BiP-binding domain, they do not directly affect α-helices mediating the physical interaction with BiP. The ambiguous missense variant, p.R92W, localizes within the N-terminal domain of the 461 amino acid SIL1 protein and p.L457P, another known pathogenic missense mutant of SIL1 affects one amino acid localized before the known ER-retention motif KDEL/KELR (17) (Figure 1A). Interestingly, *in silico* testing of pathogenicity with different platforms revealed controversial results including a probably damaging effect for all amino acid substitutions addressed in this study (Figure 1B). This result is in contrast with the observed allele frequencies in the control population and by the same token emphasizes the need to define biomarkers that can be used to evaluate the pathogenicity of *SIL1* mutations—in particular of amino acid substitutions.

### Molecular Characteristics of Selected SIL1-Variants in a Non-reducing PAGE

**Figure 2:**
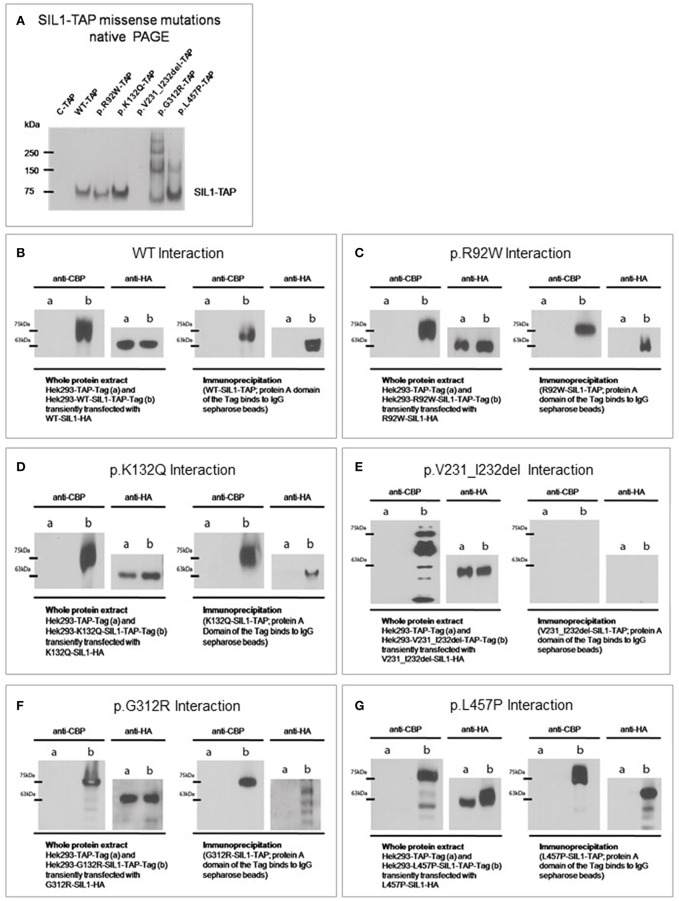
Impact of amino acid substitutions and deletions on the molecular characteristics of SIL1. **(A)** Native-PAGE of SIL1 variants reveals absence of p.V231_I232del mutant SIL1 (16 h of induced overexpression) but no differences in the migration pattern of the different missense variants of SIL1. The two known pathogenic variants p.G312R- and p.L457P present additional bands of a higher molecular weight. **(B–G)** Immunoprecipitation and subsequent immunoblot analysis toward the study of SIL1-homomerization confirmed the known SIL1-SIL1 interaction of the wildtype form of the protein **(B)** and showed no effect of the p.R92W- or p.K132Q-amino acid substitutions on this interaction **(C,D)**. Study of p.V231_I232del mutant SIL1 (6 h of induced overexpression) did not show a physical interaction of the degradation products of this known pathogenic and instable form of SIL1 **(E)**. Analysis of the two known missense mutants p.G312R- and p.L457P revealed in both cases a homomerization of the full-length mutant proteins but also an interaction of the mutant full-length protein with the respective degradation products **(F,G)**.

First, molecular characteristics of the selected SIL1-variants were addressed in a non-reducing PAGE (and concomitant blotting to a PVDF-membrane) utilizing whole protein extracts. Detection of the different variant forms of the recombinant SIL1-protein utilizing an anti-SIL1 antibody (see Table 1) revealed no major differences in the detected molecular weight of wildtype SIL1-TAP in comparison to the ambiguous p.R92W and the benign p.K132Q variants of SIL1. In contrast, no band was detectable for p.V231_I232del-mutant SIL1 (16 h of induced overexpression) suggesting instability of the aberrant protein, a finding which is in line with our previous studies on patient derived cells (6, 12). However, absence of this mutant form of the protein might also arise from a changed antigenicity of this variant product. Immunoblots of the two well-known pathogenic mutant forms, p.G312R and p.L457P, did not only show protein bands of the predicted size of the recombinant fusion-protein (SIL1-TAP) but also additional bands corresponding to a higher molecular weight which appear to be more pronounced for p.G312R (Figure 2A). The experiment was performed 6 times with similar results.

**Table 1:** All immunoblot studies were carried out at least three times.

**Antibody**	**Company**	**Dilution**
α-Beclin	Novus Biologicals: NB110-87318	1:1000
α-CBP	Thermo Fisher Scientific: PA1-847	1:1000
α-CHOP	Novus Biologicals: NB600-1335SS	1:500
α-DJ-1	Santa Cruz: sc-55572	1:750
α-DNAJB6	Genetex: GTX33160	1:500
α-ERj3	Novus Biologicals: NBP1-68477	1:500
α-FAM134B	Genetex: GTX46621	1:500
α-GAPDH	Genetex: GTX627408	1:1000
α-GRP94	Genetex: GTX103203	1:1000
α-GRP170	Genetex: GTX102255	1:1000
α-HA	Sigma Aldrich: H3663-100UL	1:500
α-IRE1	Genetex: GTX130387	1:500
α-pEif2α (Ser52)	Thermo Fisher Scientific: 44-728G	1:1000
α-PERK	Abcam: ab65142	1:500
α-POC1A	Thermo Fisher Scientific: PA5-49028	1:500
α-SIL1	Abcam: ab5639	1:100
α-SOD1	Genetex: GTX100554	1:1000
α-VCP	Genetex: GTX101089	1:1000

*List of antibodies used in the study*.

### Influence of Amino Acid Substitutions/Deletions on SIL1-SIL1-Interactions:

Given that SIL1 can act as a dimer (6), we addressed the effect of the various amino acid changes and of the deletion of two amino acids (p.V231_I232del) on the formation of SIL1-protein homomers by immunoprecipitation and subsequent immunoblot analyses. As a proof-of-principle, the well-known homomerization of SIL1 wild-type proteins was confirmed (Figure 2B). Both, the ambiguous p.R92W and the benign p.K132Q variants showed a homomerization similar to the one observed for the wild-type SIL1-TAP-SIL1-HA complex (Figures 2C,D). Analyses of the well-known pathogenic p.V231_I232del mutation revealed no interaction of the resulting degradation products in terms of the formation of toxic (proteolytic) protein complexes (Figure 2E). Study of the two-known pathogenic SIL1-proteins, p.G312R and p.L457P, showed that they can form homomeric complexes along with their degradation products (Figures 2F,G). Immunoblot-based analyses of whole protein extracts/ straight lysates confirmed the expression of the respective recombinant SIL1-variants tagged with HA or TAP in each of the respective experiments. All immunoprecipitation and immunoblot experiments were performed three times with similar results.

### Influence of Amino Acid Substitutions/ Deletions on the Fitness of the *in vitro* Models

**Figure 3:**
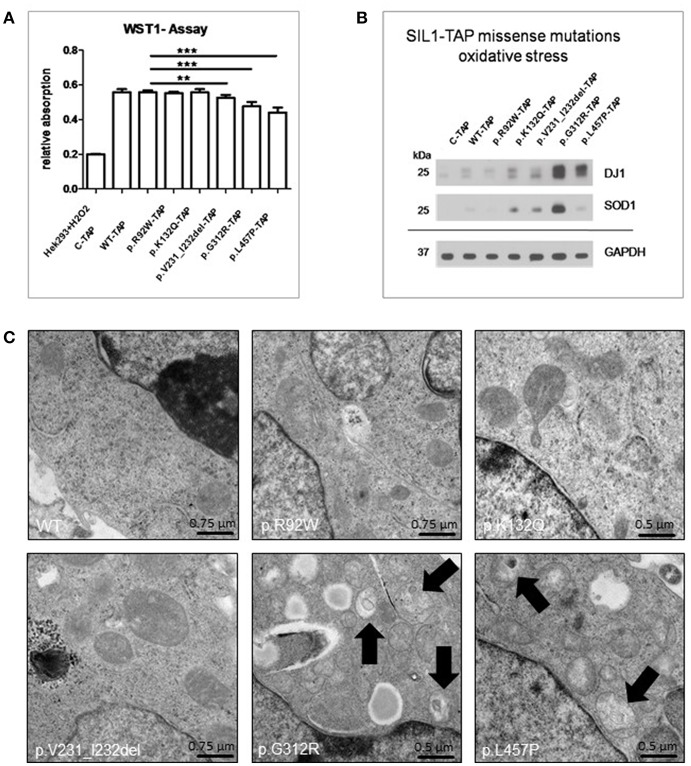
Study of fitness of Hek293 cells overexpressing SIL1 or its variant forms in the context of mitochondrial vulnerability. **(A)** ELISA-based WST-1 assay showed a statistically significant reduction in the fitness of cells overexpressing the known pathogenic variants p.V231_I232del (6 h of induced overexpression), p.G312R and p.L457P, respectively. In accordance with the instability of p.V231_I232del mutant SIL1, the fitness is more reduced in Hek293 cells overexpressing p.G312R and p.L457P mutant forms of SIL1. Hek293 cells treated with H_2_O_2_ were included as positive control showing the functionality of the assay. **(B)** Immunoblot-based studies of DJ1 (lower band) and SOD1 as two representative marker proteins for oxidative stress (related to mitochondria) showed a considerable increase of DJ1 in cells overexpressing p.G312R or p.L457P mutant SIL1. Surprisingly, SOD1 was only markedly increased in cells overexpressing p.G312R mutant SIL1, but not in cells overexpressing p.L457P mutant SIL1. GAPDH was used to demonstrate equal protein loading. **(C)** EM revealed normal mitochondrial architecture in Hek293 cells overexpressing WT-SIL1, p.R92W and p.K132Q variant SIL1 as well as p.V231_I232del mutant SIL1 but disintegration of cristae and presence of membranous electron-dense material within the mitochondrial matrix (black arrows) in cells overexpressing the two known pathogenic missense variants p.G312R and p.L457P. ^**^Statistically very significant; ^***^Statistically extremely significant.

Mitochondrial vulnerability has been repeatedly reported in MSS and woozy mouse tissues (13, 18–20), and in *in vitro* models of the disease (13, 16). As mitochondrial function is essential for cellular fitness, we examined metabolic activity of our *in vitro* models by focussing on processes related to mitochondrial activity as well as on mitochondrial morphology. The WST-1 assay (Roche) was used; in this assay the stable tetrazolium salt, WST-1, is cleaved to a soluble formazan. This conversion depends on the cellular availability of succinate-tetrazolium reductase system that belongs to the respiratory chain of the mitochondria (only active in metabolically intact cells). Thus, the amount of formazan dye formed directly correlates to the number of metabolically intact cells in the culture. Metabolic activity in WT-SIL1 cells was defined as 100% of cellular fitness. The WST-1 assay revealed no reduction in metabolic activity upon overexpression of the ambiguous p.R92W or of the benign p.K231Q variant of SIL1 (Figure 3A). Overexpression of the p.V231_I232del mutant form of the SIL1-protein (which displayed instability in our previous experiment, see above) resulted in a minor but significant decrease of the fitness (Figure 3A). In contrast, overexpression of the known pathogenic mutants p.G312R and p.L457P led to a 15–20% reduction in cellular viability compared to cells overexpressing the WT-SIL1 (or solely the TAP-Tag) suggesting an effect of their pathogenicity on cellular metabolism (Figure 3A). H_2_O_2_-treated Hek293 cells were included as a positive control for perturbed mitochondrial function based on massive oxidative stress burden (Figure 3A). The assay was carried out 4 times with similar results. Given that mitochondrial vulnerability might be connected to oxidative stress, a well-known epiphenomenon of ER-stress, levels of DJ1 and SOD1 (two proteins known to be modulated by oxidative stress burden) were studied by immunoblotting. Hek293 cells overexpressing the pathogenic missense variants of SIL1, p.G312R, and p.L457P, showed an increase in DJ1 (lower band), with a more pronounced effect in cells overexpressing p.G312R mutant SIL1. Cells overexpressing p.G312R mutant SIL1 showed a considerable increase in SOD1 protein, whereas there was no difference between the cells overexpressing the wildtype protein and those overexpressing the pathogenic p.L457P variant (Figure 3B). However, the increase of SOD1 in Hek293 cells overexpressing p.V231_I232del mutant form was comparable to the SOD1 increase in cells overexpressing the benign p.K132Q variant (Figure 3B. Notably, a parallel “trend” of abundances of DJ1 and SOD1 expression changes modulated by SIL1 variants can be observed. Prompted by the results of the WST-1 assay and by the immunoblot studies, mitochondrial-morphology was examined by electron microscopy. EM revealed normal ultrastructure of Hek293 cells overexpressing wild-type SIL1 as well as p.R92W- and p.K132Q variant forms of the SIL1 protein (Figure 3C). In contrast, Hek293 cells overexpressing the known pathogenic SIL1-missense mutations, p.G312R and p.L457P, presented with perturbed mitochondrial architecture including break-down of cristae and accumulation of electron-dense membranous material in the mitochondrial matrix most likely indicative of incipient mitophagy/ mitoptosis (21) (Figure 3C). Overexpression of the pathogenic but instable p.V231_I232del mutant form did not result in such a profound mitochondrial pathology. This might actually be due to the higher stability of the pathogenic missense mutant forms of SIL1 resulting in persistent mitochondrial stress. Pathomorphological mitochondria could be detected in 41 of 50 cells (82%) expressing the p.L457P and in 38 of 50 cells (76%) expressing the p.G312R mutant form whereas in cells expressing wildtype SIL1 or the variants p.R92W and p.K132Q, pathomorphological mitochondria were found in 10%, 18% and 14%, respectively (50 cells were analyzed for each *in vitro* model). Only 12% of Hek293 cells overexpressing the pathogenic but instable p.V231_I232del mutant displayed morphological aberrant mitochondria.

### Pattern of Cellular SIL1 Staining—Polymorphic vs. Pathogenic Forms of the Protein:

**Figure 4:**
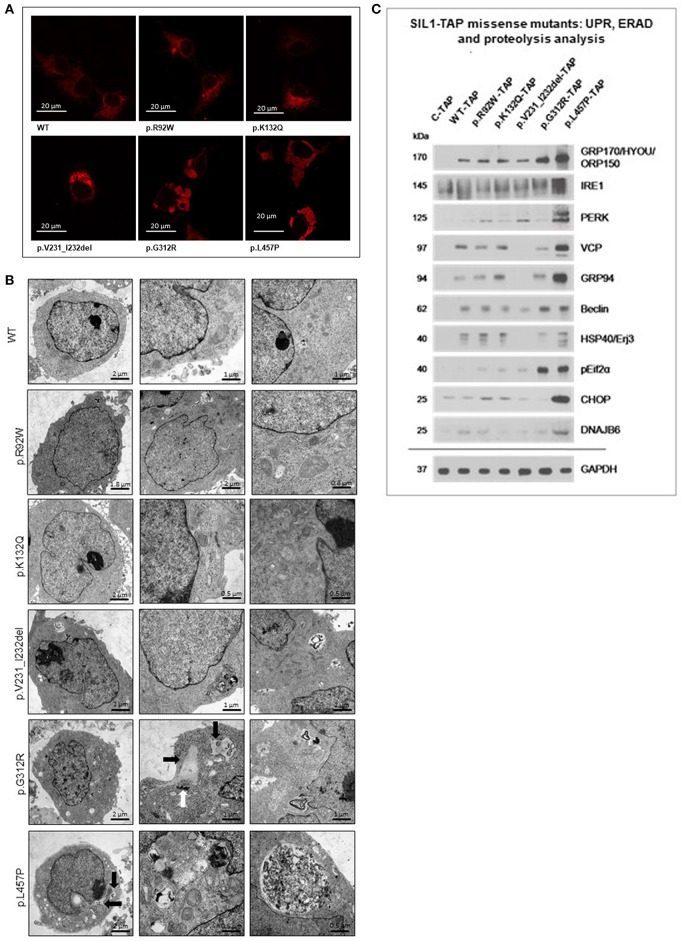
Study of cellular SIL1 immunoreactivity and presence of ER-stress in Hek293 cells overexpressing SIL1 or its variant forms. **(A)** Immunofluorescence-based investigation of the SIL1-immunoreactivity pattern in Hek293 cell revealed a reticular distribution for WT-SIL1, p.R92W and p.K132Q variant forms of SIL1 but a non-reticular rather “clumpy” immunoreactivity for p.G312R and p.L457P mutant forms of SIL1. For p.V231_I232del mutant SIL1, a reticular distribution with focal accumulations was observed. **(B)** EM revealed widened and proliferated ER in cells overexpressing the mutant forms p.G312R and p.L457P, but not in cells overexpressing WT-SIL1, p.R92W, p.K132Q, and p.V231_I232del (6 h of induced overexpression) forms of the proteins. In addition, accumulation of electron dense material could occasionally be seen in cells overexpressing p.G312R and p.L457P muatnt SIL1 (black arrows). Along this line, overexpression of these two known missense mutants of SIL1 resulted in the build-up of electron dense material (most likely corresponding to aggregated protein) in the cytoplasm as well as in (autophagic) vacuoles. The white arrow highlights an abnormal centrosome in a Hek293 cell overexpressing p.G312R mutant SIL1. **(C)** Immunoblot-based studies aimed to investigate the presence of ER-Stress by focussing on marker proteins for activation of the unfolded protein response (GRP170, IRE1, PERK, GRP94, ERj3 & pEif2α), the ER-associated degradation pathway (VCP, DNAJB6) and proteolysis (Beclin). Results revealed a considerable presence of ER-stress in Hek293 cells overexpressing p.L457P mutant SIL1 than in cells overexpressing p.G312R mutant SIL1 where solely pEif2α and Beclin are increased compared to cells overexpressing WT-SIL1. p.R92W, p.K132Q variant SIL1, and p.V231_I232del mutant SIL1 (6 h of induced overexpression) do not show remarkable changes in abundances of the stress markers compared to cells overexpressing WT-SIL1. GAPDH was investigated to demonstrate equal protein loading. The differences in expression of these markers between C-TAP and WT-SIL1 overexpressing Hek293 cells have been described previously (12).

Immunofluorescence studies of our Hek293 *in vitro* models overexpressing the different forms of the SIL1 protein in an inducible fashion were performed to address the question if the pattern of SIL1-TAP-immunoreactivity allows to differentiate between polymorphic variants and pathogenic mutations. Indeed, cells overexpressing WT-SIL1 in comparison to those overexpressing the two known pathogenic missense forms (p.G312R and p.L457P) showed significant differences regarding the pattern of immunoreactivity. WT-SIL1 was distributed in a reticular network in accordance with a regular ER-localization, whereas p.G312R- and p.L457P mutant SIL1 showed an altered non-reticular immunoreactivity pattern (Figure 4A). In contrast, p.V231_I232del mutant SIL1 (after 6 h of induced overexpression) showed a reticular distribution comparable to WT-SIL1 and the polymorphic p.K132Q form but with some focal accumulations (Figure 4A). Notably, immunoreactivity of p.R92W variant SIL1 does not show the same subcellular distribution as the p.G312R and p.L457P mutants and rather presents as a reticular network as observed for WT-SIL1 and p.K132Q but with some minor focal accumulations.

### Influence of Amino Acid Substitutions/Deletions on ER-Morphology and Function in our *in vitro* Models

To follow up on the immunofluorescence studies described above, EM was performed. ER in Hek293 cells overexpressing WT-, p.R92W and p.K132Q versions of SIL1 showed a regular tubular structure. In cells overexpressing p.G312R and p.L457P mutant SIL1, the ER showed focal widening, occasionally associated with accumulation of electron dense material most likely corresponding to protein aggregates within the widened ER (black arrows in Figure 4B). In addition, cells overexpressing these two known-pathogenic mutants exhibited autophagic vacuoles that were often filled with membranous and granular osmophilic material (Figure 4B). Deposits of osmophilic material (mostly localized to autophagic vacuoles) were also detected in Hek293 cells with short-term (6 h) overexpression of p.V231_I232del mutant SIL1 (Figure 4B).

Prompted by our ultra-structural findings, we next investigated the presence of ER-stress upon overexpression of wildtype SIL1 as well as the 5 different variants at the molecular level by immunoblotting by focussing on the abundances of 10 proteins modulating the unfolded protein response (UPR) and the ER-associated degradation pathway (ERAD). In accordance with our previous published data (12), overexpression of wildtype SIL1 results in the increased expression of these factors compared to cells overexpressing solely the TAP-Tag (Figure 4C). This in turn also explains the altered expression of these factors in Hek293 cells with long-term overexpression of the instable p.V231_I232del mutant form. However, overexpression of neither the polymorphic p.K132Q nor of the ambiguous p.R92W variant led to a sustained increase of the UPR-/ ERAD-related proteins compared to cells overexpressing the wildtype form of SIL1 (Figure 4C). Overexpression of p.G312R resulted in an increased expression of GRP170 and Beclin-1 as well as forced phosphorylation of eIF2α compared to cells overexpressing the TAP-tag only, the wildtype variant of SIL1 or the p.R29W and p.K132Q variants, respectively (Figure 4C). The overexpression of p.L457P mutant SIL1 even resulted in elevated abundance of all investigated UPR-/ ERAD-proteins except HSP40/ ERj3 (Figure 4C).

### Influence of Amino Acid Substitutions/Deletions on SIL1-Protein-Binding and Subsequent Morphological Effects in our *in vitro* Models

**Figure 5:**
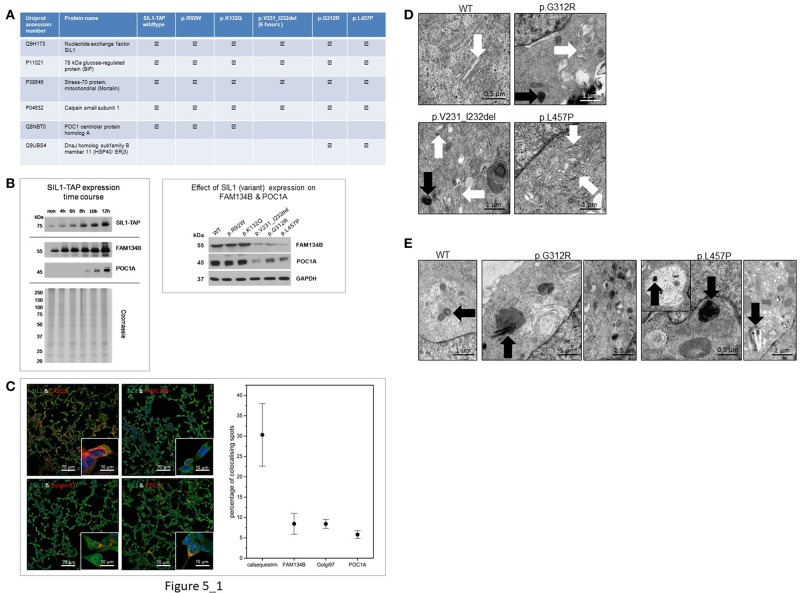
Study of SIL1-interactome and impact of interaction on subcellular morphologies. **(A)** Table presenting SIL1-protein interactions identified by tandem-affinity-purification and subsequent mass spectrometry in an unbiased fashion: whereas WT-SIL1 and its (polymorphic and pathogenic) variants interact with BiP, Mortalin and the small subunit of Calpain-1, solely WT-SIL1, p.R92W and p.K132Q variant forms of SIL1 precipitated POC1A. In contrast, solely p.G312R and p.L457P mutant SIL1 precipitated ERj3, a known BiP-binding protein controlled by ER-stress. the detection of SIL1—as shown in the table presented—refers to the identification/ detection of tryptic peptides unique for the SIL1 protein in the mass spectrometric analysis. The peptides identified for the respective SIL1-variant arise from the overexpression of same ones and show the reliability of the combined IP-LC-MS/MS data. **(B)** Immunoblot-based study of FAM134B and POC1A revealed increased expression of both proteins upon elevation of the cellular SIL1 level. Coomassie-staining was carried out to demonstrate equal protein loading (left panel). Results of expression studies in Hek293 cells expressing different mutant forms of the protein show a clear influence of the pathogenicity of SIL1-mutations on the abundance of the FAM134B and POC1A proteins (right panel). **(C)** Results of our co-immunofluorescence studies focussing on SIL1 localizations revealed co-localizations of SIL1 with CASQ1 (calsequestrin), golgin-97, FAM134B, and POC1A. Representative overviews of cells are shown for each respective co-localization study which have also been used for the quantifications of co-localizations (right diagram). **(D)** EM of Golgi morphology revealed a regular architecture in Hek293 cells overexpressing WT-SIL1, but proliferated and dispersed Golgi with widened terminal cisternae (white arrows) and vesicle accumulations in cells overexpressing p.G312R or p.L457P mutant SIL1. In cells overexpressing p.V231_I232del mutant SIL1, solely a mild pathology of the Golgi apparatus (widened cisternae indicated by white arrows) could be observed. **(E)** EM of centrosome morphology revealed a regular architecture in Hek293 cells overexpressing WT-SIL1, but proliferated, multiplicated and dispersed centrosomes in cells overexpressing the pathogenic missense mutant pG312R or p.L457P (black arrows). Remarkably, in cells overexpressing these two known pathogenic forms of SIL1, a mis-localization of abnormal centrosomes to lysosomes (black arrows in pictures 2 and 4) or to vacuoles (black arrow in the inset of picture for as well as in picture 5) was observed.

To define another read-out measure enabling the robust evaluation of the pathogenicity of amino acid changes and deletions in *SIL1*, tandem-affinity-purification followed by mass spectrometric analysis of the different TAP-tagged variants of the SIL1 protein was performed. Overexpression of p.V231_I232del mutant SIL1 was induced by doxycycline treatment for 6 h to study binding partners prior degradation of this instable pathogenic SIL1 protein. Results of this screening approach are presented in Figure 5A and show that all variants of the SIL1-protein bind to BiP, Mortalin and the small subunit of Calpain-1. Moreover, our protein-interaction studies revealed a precipitation of ERj3 along with the two pathogenic mutants of SIL1, p.G312R, and p.L457P, but not with the two variants p.R92W and p.K132Q or the wildtype form of SIL1. Remarkably, our unbiased studies focusing on interaction of different forms of SIL1 to other proteins revealed that the wildtype protein as well as the p.R92W and p.K132Q variants are precipitating with POC1A whereas immunoprecipitation of p.G312R and p.L457P mutant SIL1 along with their interactors and subsequent mass spectrometry studies did not result in the identification of POC1A as a binding partner. Given that mutations in *POC1A* are causative for SOFT (short stature, onychodysplasia, facial dysmorphism, and hypotrichosis) syndrome (22) and short stature is also a clinical finding in MSS patients, a regulatory effect of abundances of the SIL1-wildtype protein on level of POC1A was confirmed by immunoblot studies. Our findings highlight an effect of cellular SIL1 level on POC1A protein abundance (Figure 5B, left). *POC1A* encodes for a protein linking centrosomes to Golgi assembly and function, and recessive mutations affecting this gene result in perturbed Golgi structures and cytosolic vesicle accumulations, in addition to abnormal multipolar spindles *in vitro* (22, 23). This known POC1A function along the mentioned pathological findings resulting from mutations in the corresponding gene prompted us to also study the level of FAM134B, a newly identified cis-Golgi protein with a pivotal role in neuronal function and survival (24) in relation to the expression of SIL1 wildtype protein as well as the morphology of Golgi and centrosomes. Indeed, immunoblot studies of FAM134B showed a beneficial effect of SIL1 expression on the abundance of this Golgi-protein (Figure 5B, left). Results of expression studies in Hek293 cells expressing different mutant forms of the protein show a clear influence of the pathogenicity of SIL1-mutations on the abundance of the FAM134B and POC1A proteins (Figure 5B, right).

Prompted by our SIL1-protein interaction findings suggestive for a localization of SIL1 also to the Golgi-apparatus, further immunofluorescence studies have been performed and revealed an expected co-localization of SIL1 with calsequestrin (CASQ1; a known ER-marker protein) and additionally co-localizations with golgin-97 (a known marker protein of the Golgi apparatus) as well as with FAM134B and POC1A. Quantification of these co-localizations revealed that the excessive proportion of the SIL1 protein localizes to the ER but almost equal co-localization with golgin-97, FAM134B and POC1A indicates a minor localization of SIL1 also within the Golgi apparatus (Figure 5C).

Results of further ultra-morphological studies focussing on the integrity of Golgi and centrosomes revealed proliferated and dispersed Golgi cisternae in Hek293 cells overexpressing p.G312R or p.L457P but not in cells overexpressing the wildtype form of the protein (Figure 5D). Whereas, neither the polymorphic p.K132Q nor the ambiguous p.R92W variant of SIL1 presented with changes in Golgi architecture compared to cells overexpressing WT-SIL1 (data not shown), Hek293 cells with short-term overexpression of p.V231_I232del showed only a very mild widening of Golgi cisternae (Figure 5D). Interestingly, altered Golgi structures could frequently be detected adjacent to abnormal centrosomes as exemplified for p.V231_I232del and p.G312R in Figure 5D (black arrows). Along this line, overexpression of the pathogenic forms of the SIL1 protein in Hek293 cells repeatedly resulted in disintegration of centrosome architecture including centriole-multiplications and -proliferations as well as mis-localization of centrioles to lysosomes and vacuoles (Figure 5E).

## Discussion

On a general note, the need of suitable *in vitro* systems allowing a reliable testing of ambiguous gene mutations potentially affecting stability and localization of the corresponding protein is crucial in the cases that patient-derived tissue is not available to perform verification studies. Here, using MSS as a paradigmatic rare neurogenetic disease, we demonstrate that Hek293 cells overexpressing different mutant forms of the SIL1 protein are suitable to evaluate the pathogenicity of SIL1 mutations such as missense variants and in-frame-amino acid deletions. MSS has been selected for our studies as:

- The phenotype can present with prominent additional clinical features or characteristic features of the “clinical triad” can be absent thus complicating the evaluation of detected variants in terms of phenotype-causative mutations
- Missense mutations may have a detrimental effect on the clinical presentation (10)
- Detection of new *SIL1* sequence variants is ever increasing in number thus indicating the need to classify their pathogenicity.

Indeed, the combination of different biochemical, morphological and functional studies utilizing Hek293 cells overexpressing different variants of SIL1 of known and ambiguous pathogenicity allowed to introduce a system enabling the *in vitro*-based evaluation of *SIL1* variants leading to amino acid substitutions or deletions: results of our SIL1-protein studies in a non-reducing PAGE suggest that although the pathogenic variants do not cause essential changes in the behavior of the main SIL1 band, bands of higher molecular weight occur which most likely correspond to aberrant SIL1 protein complexes. The absence of these bands under reducing conditions (data not shown) indicates that these aberrant complexes are soluble. Hence, the presence of additional (higher molecular weight) SIL1 bands in a non-reducing PAGE might serve as a read-out measure to evaluate the pathogenicity of amino acid substitutions in SIL1. The absence of these bands in immunoblots of WT-SIL1, p.K132Q and p.R92W variants of SIL1 suggest that p.R92W is a benign variant rather than a pathogenic mutation.

In addition, studies focussing on the influence of amino acid substitutions/ deletions on SIL1-SIL1-interactions provided a new outcome measure toward the evaluation of the pathogenicity of SIL1 variants: given that degradation products of the p.V231_I232del, the p.G312R and the p.L457P mutant forms of SIL1 can be identified utilizing the straight protein lysates of the SIL1-SIL1 interaction experiment and anti-HA/TAP antibodies (detecting the respective Tags at the C-terminus of the recombinant proteins) a N-terminal initiated degradation of these mutant forms of the protein is suggested. The presence of degradation products for p.G312R and p.L457P mutant SIL1 (Figures 2F,G) in combination with the detected higher molecular weight bands for these forms as presented in Figure 2A suggests formation of protein aggregates and breakdown. A comparison of the degradation of the variant forms of SIL1 with known pathogenic character suggests a much higher instability of SIL1 exhibiting deletion of two amino acids compared to the two forms with C-terminal substitutions. Taken together, this assay shows that precipitation of degradation products might be indicative for a pathogenic character of amino acid changes in SIL1.

Given that mitochondria display a considerable vulnerability in the etiology of MSS (13, 18–20), WST-1 assay has been performed to investigate mitochondrial activity in our MSS *in vitro* models and results suggest a benign character of the p.R92W and p.K132Q amino acid changes in SIL1 whereas the pathogenic character of the p.G312R and p.L457P missense mutants could be confirmed. The mild reduction of the cellular fitness in cells overexpressing the instable p.V231_I232del mutant form of SIL1 (while cells still also express the endogenous SIL1 protein) might arise from impairment of cellular processes until degradation of the mutant recombinant protein is fully achieved. Findings of this assay suggest a reduced metabolic activity as a cellular biomarker or read-out measure of pathogenicity of *SIL1* mutations. As the expression of pathogenic SIL1 variants also impacts on morphological integrity of mitochondria, presence of oxidative stress and perturbed mitochondrial architecture might represent further useful read-out measures for the *in vitro* evaluation of pathogenicity of overexpressed *SIL1* mutations that are associated with a loss of function rather than a loss of protein. However, the less pronounced oxidative stress burden in the case of overexpression of p.L457P mutant SIL1 (Figure 3B) suggests that analysis of oxidative stress should not serve as a mere measure to evaluate the pathogenic character of SIL1 missense variants.

To address cellular SIL1 immunoreactivity and distribution as a further measure to evaluate pathogenicity of SIL1 amino acid substitutions and deletions immunofluorescence studies have been carried out and -in comparison to the Hek293 cells over expressing the SIL1 wildtype protein—altered immunoreactivity could be observed for p.G312R and p.L457P and to a minor degree for p.V231_I232del overexpressing cells but not for cells overexpressing p.R92W or p.K132Q variants of SIL1. As a pathomorphological immunoreactivity-pattern has already been demonstrated for p.L457P mutant SIL1 in COS7 cells (14), the recapitulation in Hek293 cells confirms the suitability of these cells to investigate pathogenicity of *SIL1* mutations. Moreover, a similar pattern of immunoreactivity has been shown *in vitro* upon overexpression of a pathogenic form of *SIL1* caused by a deletion of thymidine at base-pair position 1366 of the corresponding gene (17). Consequently, our immunofluorescence findings confirm the pathogenicity of p.G312R mutant SIL1. One might speculate that the small focal accumulations of the p.V231_I232del mutant form of SIL1 represent early stages of degradation of the mutant protein, an assumption that is in line with the detection of degradation bands upon short-term overexpression (Figure 2B) and the absence of the protein upon long-term overexpression (Figure 2A). As immunofluorescence studies of p.R29W did not reveal pathological changes compared to the cells overexpressing the wildtype or the p.K132Q variant form of SIL1, the combined results support the notion that p.R92W is a polymorphic variant rather than a pathogenic mutation and also indicate that the pattern of SIL1 immunoreactivity might serve as another cellular biomarker or read-out measure allowing the evaluation of pathogenicity of SIL1 amino acid substitutions.

The altered distribution and aggregation of p.G312R and p.L457P mutant forms of SIL1 prompted us to focus on subcellular ultrastructural alterations suggestive for protein aggregation and proteolysis. Indeed, electron microscopic studies revealed the presence of vacuoles in Hek293 cells overexpressing these two pathogenic missense variants of SIL1. One might assume that these vacuoles result from impaired ER-function and thus protein processing resulting in accumulation of osmophilic material which could also be observed in the cytoplasm (not localized within vacuoles; white arrows in Figure 4B). As the p.V231_I232del pathogenic mutation is instable, protein aggregates and vacuoles identified by electron microscopy might correlate to the degradation of the mutant SIL1 protein. Importantly, build-up of electron-dense aggregates and altered ER morphology have also been described in immortalized lymphoblastoid cells from patients [including heterozygosity for p.V231_I232del and p.G312R, (6, 13] as well as in Hek293 cells with depleted SIL1 expression (16) but also in MSS-patient derived fibroblasts (25). This in turn confirms the suitability of our generated *in vitro* overexpression models to evaluate the pathogenicity of SIL1 mutations on the ultra-structural level. However, results of our immunoblot-studies focussing on ER-stress and activation of proteolysis suggested a more profound ER-vulnerability and proteolytic activation in Hek293 cells overexpressing the p.L457P-mutant form of SIL1 and furthermore again suggest that the p.R92W is in fact a polymorphic missense variant of SIL1. Based on the relatively low effect of the overexpression of p.G312R (showing a detrimental effect on oxidative homeostasis; see above) on ER-homeostasis and proteolysis compared to overexpression of p.L457P, the necessity of parallel investigations of different read-out measures to draw a meaningful conclusion regarding the pathogenicity of *SIL1* variants is indicated.

SIL1-interactome has been addressed as a potential further measure to evaluate the pathogenicity of amino acid changes in SIL1 and results showed that all variants of the SIL1-protein investigated in this study bind to BiP, Mortalin and the small subunit of Calpain-1. In this context it is important to note that an ER localization of Mortalin which shows a high homology to BiP has already been described (26). Given that Calpain-1 acts as a non-lysosomal thiol-protease catalyzing proteolytic cleavage of substrate proteins, its binding to all five variants of SIL1 as well as to WT-SIL1 might result from protein overexpression and serves toward the elimination of excessive level, an assumption supported by our previous findings in Hek293 cells with WT-SIL1 overexpression (12). As BiP represents a well-known binding partner of SIL1 but also acts as a major chaperone in the UPR facilitating the re-folding of misshaped proteins, it is difficult to evaluate whether the interaction of BiP with the different variants of SIL1 is physiological or a result of an attempt to warrant a native folding of the protein in case of the binding to the pathogenic forms of SIL1. Notably, localization of p.V231_I232del- and p.G312R-amino acid changes in the BiP-binding domain of SIL1 (Figure 1A) are suggestive for a binding of BiP to these SIL1-forms (or degradation products) toward re-folding and antagonization of build-up of (toxic) protein aggregates. This hypothesis is further supported by the fact that DNAJB11/ERj3 serves as another BiP co-chaperone (stimulating ATPase activity) by direct binding to both unfolded ERAD-substrates and nascent unfolded peptides, but dissociates from the BiP-unfolded protein complex before folding is completed (27) and our molecular observation of ERj3 binding to p.G312R and p.L457P mutant SIL1 but not to WT-SIL1, and the p.R92W and p.K132Q variants. Thus, it seems plausible that the functional ERj3-BiP complex is recruited to the pathogenic missense variants based on pathophysiological circumstances. On a general note, this finding moreover suggests that ERj3 binding to SIL1 or rather the SIL1-BiP complex might serve as a molecular measure/ biomarker to evaluate the pathogenicity of stable SIL1 missense variants. However, results of our immunoblot studies of ER-stress related proteins did not show elevated ERj3 level in Hek293 cells overexpressing p.G312R and p.L457P mutant SIL1 compared cells overexpressing the wildtype SIL1 protein (Figure 4C) indicating that cellular available ERj3 pools are re-located to the two pathogenic mutant forms of SIL1. Mass spectrometry-based analysis of SIL1-interacotme moreover revealed that the wildtype protein as well as the p.R92W and p.K132Q variants bind to POC1A. Interestingly, immunoprecipitation of the p.G312R and p.L457P forms of SIL1 along with their interactors did not result in the identification of the binding to POC1A. This finding defines the interaction of POC1A with SIL1 variants as further read-out measure to evaluate the pathogenicity of amino acid substitutions and deletions in *SIL1* and by the same token underlines our previous findings suggesting that the p.R92W variant form of SIL1 is in fact a polymorphic variant rather than a pathogenic mutant of the BiP co-chaperone. On a more general note, our immunoprecipitation data support the previous description of an interaction of POC1A with the SIL1-BiP machinery as identified by BioPlex 2.0 [Biophysical Interactions of ORFEOME-derived complexes; (28)].

Prompted by the fact POC1A mutations are causative for a phenotype defined by short stature, onychodysplasia, facial dysmorphism, and hypotrichosis (SOFT syndrome; 22) and short stature is also a recurrent clinical finding in MSS patients, effect of SIL1 expression (wildtype and variant forms) has been studied and highlighted an increasing effect of elevated SIL1 protein level on POC1A protein abundance (Figure 5B). Same effect could be observed for p.R92W and p.K132Q but not p.G312R, p.L457P or p.V231_I232del mutant forms of SIL1. This effect indicates that the molecular interaction between the two proteins might also be of relevance for the clinical manifestation of MSS upon loss of (functional) SIL1. Given that POC1A links centrosomes to Golgi assembly and function we further studied level of FAM134B, a newly identified cis-Golgi protein with a pivotal role in neuronal function and survival (24) in relation to the expression of SIL1 wildtype protein. Here same results as for POC1 could be obtained (Figure 5B) suggesting an impact of SIL1 expression on the Golgi apparatus, an assumption which accords with the newly identified SIL1-POC1A interaction and the results of our further immunofluorescence studies not only showing a co-localization of SIL1 with POC1A and FAM134B but also with golgin-97, a known Golgi-marker protein (Figure 5C). Results of these immunofluorescence-based localization studies in combination with our immunoprecipitation findings also suggest that SIL1 localizes (although to a minor degree) to subcellular compartments beyond the ER. However, to follow the hypothesis that pathogenic SIL1 variant significantly impact on function and structure of centrosome and the Golgi apparatus, ultra-structural studies focussing on their integrity have been carried out and revealed proliferated and dispersed Golgi cisternae as well as centriole-multiplications and -proliferations in Hek293 cells overexpressing the known pathogenic forms of SIL1 but not in cells overexpressing the wildtype or the p.R92W or p.K132Q forms of the protein (Figures 5D,E). These pathomorphological findings in turn further support the concept of an impact of SIL1 on Golgi and centrosome function and integrity and by the same token define changes in Golgi and centrosome structures as further read-out measures enabling the evaluation of the pathogenicity of *SIL1* mutations. In this context, it is important to note that depletion of SIL1 expression in Hek293 cells could be linked to vulnerability of the Golgi apparatus on both the structural and the biochemical level (16).

## Conclusions

Generation and subsequent functional, biochemical and morphological phenotyping of our *in vitro* models overexpressing different (benign and pathogenic) forms of the SIL1 protein allowed to define cellular markers to evaluate the pathogenicity of SIL1-mutations, an aspect of significant importance for genetic counseling and genotype-phenotype correlations. Suitable assays and respective read-out measures (in terms of cellular biomarkers for pathogenicity of changes in SIL1 amino acid composition) are summarized in Table 2.

**Table 2:** Overview of cellular markers and respective analytical approaches toward evaluation of pathogenicity of SIL1-mutants.

**Test**	**Cellular marker of pathogenicity**
Native and reducing PAGE with subsequent Western-Blot of SIL1 protein	Higher and lower molecular weight bands as signs of protein aggregates (in native PAGE) and degradation products
WST-1 viability assay	Reduced viability
Immunofluorescence of SIL1-protein	Non-reticular pattern of immunoreactivity
Electron microscopy	Abnormal mitochondria, vacuoles & protein aggregates, dispersed Golgi, disintegrated centrosomes
SIL1-interactome	Loss of interaction with POC1A and binding to DNAJB11/ ERj3
Immunoblot analysis of cellular stress markers	Oxidative- and/ or ER-stress burden
SIL1-SIL1 homomerization	Occurrence of degradation products of pathogenic mutant SIL1-protein which do not present rapid degradation

## Author Contributions

AR and JW conceptualized and designed the study, corrected the manuscript and supervised the experimental work. SB generated the different expression constructs used in this study. TL assisted CG in the generation of the stable-inducible cell Hek293 cell lines. DH performed the mass spectrometric analysis of the SIL1-interactome. AT performed the *in silico-*based analysis of pathogenicity of selected variants. UM performed the immunofluorescence-based co-localization studies of SIL1. CG performed all other experiments and drafted the first version of the manuscript. RH and BM gave expert opinion regarding the mitochondrial findings.

### Conflict of Interest Statement

The authors declare that the research was conducted in the absence of any commercial or financial relationships that could be construed as a potential conflict of interest.
